# Saliva enhances infection of gingival fibroblasts by herpes simplex virus 1

**DOI:** 10.1371/journal.pone.0223299

**Published:** 2019-10-03

**Authors:** Yi Zuo, J. Charles Whitbeck, Gabriel J. Haila, Abraham A. Hakim, Paul W. Rothlauf, Roselyn J. Eisenberg, Gary H. Cohen, Claude Krummenacher

**Affiliations:** 1 Department of Microbiology, School of Dental Medicine University of Pennsylvania, Philadelphia, Pennsylvania, United States of America; 2 Department of Chemistry and Biochemistry, Rowan University, Glassboro, New Jersey, United States of America; 3 Department of Biological Sciences, Rowan University, Glassboro, New Jersey, United States of America; 4 Department of Pathobiology, School of Veterinary Medicine, University of Pennsylvania, Philadelphia, Pennsylvania, United States of America; 5 Department of Molecular and Cellular Biosciences, Rowan University, Glassboro, New Jersey, United States of America; Cornell University, UNITED STATES

## Abstract

Oral herpes is a highly prevalent infection caused by herpes simplex virus 1 (HSV-1). After an initial infection of the oral cavity, HSV-1 remains latent in sensory neurons of the trigeminal ganglia. Episodic reactivation of the virus leads to the formation of mucocutaneous lesions (cold sores), but asymptomatic reactivation accompanied by viral shedding is more frequent and allows virus spread to new hosts. HSV-1 DNA has been detected in many oral tissues. In particular, HSV-1 can be found in periodontal lesions and several studies associated its presence with more severe periodontitis pathologies. Since gingival fibroblasts may become exposed to salivary components in periodontitis lesions, we analyzed the effect of saliva on HSV-1 and -2 infection of these cells. We observed that human gingival fibroblasts can be infected by HSV-1. However, pre-treatment of these cells with saliva extracts from some but not all individuals led to an increased susceptibility to infection. Furthermore, the active saliva could expand HSV-1 tropism to cells that are normally resistant to infection due to the absence of HSV entry receptors. The active factor in saliva was partially purified and comprised high molecular weight complexes of glycoproteins that included secretory Immunoglobulin A. Interestingly, we observed a broad variation in the activity of saliva between donors suggesting that this activity is selectively present in the population. The active saliva factor, has not been isolated, but may lead to the identification of a relevant biomarker for susceptibility to oral herpes. The presence of a salivary factor that enhances HSV-1 infection may influence the risk of oral herpes and/or the severity of associated oral pathologies.

## Introduction

The highly prevalent herpes simplex virus 1 (HSV-1) is the etiologic agent of oral herpes. In 2015–2016, 48% of American adults were seropositive for HSV-1 [1]. HSV-1 primary infection causes gingivostomatitis, which can go unnoticed or cause mucosal ulcerations of various severity [2]. The related HSV-2 is the main agent of herpes genitalis but only rarely causes oral disease [3]. After replication in oral epithelial cells, HSV-1 spreads to innervating sensory neurons, where it establishes latency [4]. This latent phase is punctuated by reactivation episodes during which viral replication in epithelia generates mucocutaneous lesions (cold sores)[2]. Importantly, HSV-1 reactivation often occurs asymptomatically and leads to frequent unnoticed shedding from the oral mucosa [5–7]. For instance, in a cohort of 8 immunocompetent individuals evaluated during 5 consecutive weeks, asymptomatic reactivation was observed at sites throughout the oral cavity at a rate of 27.1% (65/240days) [5]. The variability in frequency of HSV-1 reactivation and severity of herpes diseases is thought to be related to the host immunogenetic factors [8]. Although particular genetic markers have been associated with risks of herpes simplex encephalitis [9], biomarkers associated with risks or severity of oral herpes have not yet been identified [10].

Herpesviruses have been found in healthy and pathological oral tissues, in particular they are associated with periodontal disease (PD)[11]. About 47% of American adults suffer from PD [12]. Subgingival colonization by Gram negative facultative and anaerobic bacteria plays a major role in the development of PD [13]. Interestingly, HSV-1 has been detected in lesions during chronic and aggressive periodontitis [14–17]. The role of HSV-1 in PD pathology remains unclear but several studies associated it with increased severity of lesions [18–20]. Since HSV-1 infection interferes with immune regulators, it may aggravate PD by causing local immunosuppression and inflammation [21, 22].

Oral keratinocytes and epithelial cells, which comprise the main sites of lytic replication during primary and secondary lytic infections, are highly susceptible to HSV-1 infection *in vitro* [23]. In contrast, gingival fibroblasts, which are normally not exposed in the oral mucosa are less efficiently infected *in vitro* [24, 25]. Infection of intact oral epithelia *ex vivo* is inefficient and depends on access to entry receptors on basal keratinocytes [23]. Nectin-1 and HVEM are the main HSV receptors on various oral cells [23, 25]. Interaction of nectin-1, HVEM or 3-O-sulfated heparan sulfate, with HSV glycoprotein D (gD) is an essential step in entry [26, 27]. Receptor-triggered conformational changes in gD initiate the activation of gH/gL, which in turn activates gB to fuse the viral envelope with a cell membrane [28, 29]. In addition, binding of gD to nectin-1 or HVEM induced virus endocytosis in certain cell types [30–32]. Nectin-1 is an adhesion molecule accumulating at adherens junctions at the basolateral side of epithelial cells [33] and junction disruption increases infectivity [23, 34–36]. Apical infection of oral epithelial cell is inefficient and, although local wounding can favor access to basolateral receptors, the number of infected cells around wounding sites remained limited, suggesting that further tissue damage may be required for viral invasion [23]. Clearly, receptor availability and access strongly influence susceptibility of cells to HSV infection [37, 38]. Other determinants of cell susceptibility or permissivity to HSV-1 and -2 infection are less clearly defined.

Saliva is a complex fluid involved in digestion, protection of teeth, wound healing, taste recognition and even speaking [39, 40]. Furthermore, saliva plays a major protective role against pathogens. Salivary components have anti-microbial and/or antiviral activity [41–44]. In particular, saliva can inactivate HSV-1 to protect oral cells [45–47]. Saliva is considered an important resource for diagnostic and disease biomarkers [48].

Here we report that primary diploid gingival fibroblasts became increasingly susceptible to HSV-1 infection after being exposed to human salivary extract. Interestingly, B78H1 mouse melanoma cells, which are resistant to HSV-1 and -2 due to a lack of entry receptors, became infected after exposure to salivary proteins. Entry into saliva-stimulated B78H1 cells could be blocked by anti-HVEM antibody. Although the cellular response to saliva has not been fully characterized, it appears to affect susceptibility to infection by influencing the level of virus entry. The saliva activity was linked to large complexes of salivary glycoproteins, possibly involving immunoglobulin A (IgA). However, prior HSV-1 or -2 infection of the donor appears not to be required for this activity, thus indicating that the potentially involved IgA’s are not elicited by HSV. Importantly, we observed a broad variation in the intensity of salivary activity, which was confined to only a subset of donors. Further studies will tell whether this variation in saliva activity correlates with an increased risk of HSV-1 oral infection and/or severity of oral symptoms.

## Material and methods

### Cells, viruses and antibodies

AG09319 cells (Coriell Institute) are diploid gingival fibroblasts, grown in DMEM, 10% fetal calf serum (FCS) and penicillin/streptomycin. B78H1 mouse melanoma cells are resistant to HSV-1 and -2 infection due to the absence of receptors, and C10 cells are derived from B78H1 to express human nectin-1 [49]. B78H1 cells were grown in DMEM with 5% FCS and penicillin/streptomycin (0.5 mg/ml G418 was added for C10 cells). HSV-1 KOStk12 and HSV-2(333)gJ- reporter viruses were generously provided by P.G. Spear [50–52]. Viruses were grown and titered on Vero cells, and purified as described previously [53]. The anti-HVEM-receptor rabbit serum R140 and the anti-nectin-1 mouse monoclonal antibody (mAb) CK41 were described previously [54, 55].

### Saliva collection

This study was approved by the internal review board (IRB) of the University of Pennsylvania (protocol # 812549) and by the IRB from Rowan University (protocol # Pro2014000104). Written informed donor consent was obtained. Unstimulated saliva was collected by having subjects expectorate in a collection tube. Donors were requested to brush their teeth and abstain from eating and drinking for at least 1h prior to donating 20–40 ml saliva. Samples were not collected when a subject had evidence of active HSV infection. Samples were stored at 4°C (max. 24h) or frozen (-80°C). Samples were centrifuged (3500 rpm, 30 min) to remove solid debris. Clarified saliva was passed through a 5 μm filter and sterilized through a 0.22 μm filter. Saliva was then concentrated by centrifugation through a 30 kDa cutoff filter (Amicon) and the retentate was collected. During this process, concentrated samples were buffer exchanged 3 times with 10 mM sodium phosphate buffer at pH = 7.25 (if used for fractionation) or PBS. The final retentate was used for cell stimulation unless specified otherwise. Protein concentration was determined using a Coomassie colorimetric assay. All saliva samples were stored at -80°C.

### Saliva fractionation

Processed saliva samples (30 kDa retentate) were diluted in 10 mM phosphate buffer, pH 7.25 prior to loading onto a MonoS ion exchange column (BioRad). The flow-through was eluted by gravity. The column was washed with 10 mM phosphate buffer pH 7.25 and bound proteins were eluted with increasing concentrations of NaCl in 10 mM phosphate buffer (1 ml fractions). The MonoS column flow-through was loaded onto a MonoQ ion exchange column (BioRad). Again, the flow-through was collected and the column washed with the phosphate buffer. Bound proteins were eluted by increasing NaCl concentrations in 10 mM phosphate buffer pH 7.25. Salt-containing fractions were concentrated and buffer exchanged against 10 mM phosphate buffer pH 7.25. The 250mM NaCl fraction was loaded onto a Superdex200 column (GE Healthcare) for size exclusion chromatography using 10 mM phosphate buffer pH 7.25. 0.5 ml fractions were collected, pooled and concentrated via centrifugal filtration. Protein content was separated by SDS-PAGE and revealed by Coomassie or silver staining as previously described [56].

### Saliva treatments

**1.** Heat-denaturation: Samples were boiled for 5 min and rapidly cooled on ice. **2.** Proteinase-K treatment: Proteinase-K agarose (P9290, Sigma-Aldrich) was hydrated in 10mM sodium phosphate buffer at pH 7.0. Fifty μl of packed beads were added to 100 μl saliva proteins at 6.2 mg/ml and incubated for 4h at 37°C. The supernatant was diluted in DMEM to an equivalent of 1 mg/ml protein concentration for cell stimulation. **3.** Jacalin capture: Jacalin agarose (Pierce/Thermo) was hydrated in PBS. Fifty μl of packed beads were added to 100 μl saliva proteins at 6.2 mg/ml and incubated for 4h at 4°C. The supernatant was diluted in DMEM to an equivalent of 1 mg/ml protein.

### Cell stimulation and infection

Subconfluent cells in 96-well plates were incubated with salivary proteins diluted in growth medium for 48-72h. Stimulation medium was removed and cells were washed with medium prior to addition of virus. Saliva-treated or mock-treated cells were exposed to sucrose-purified HSV-1 KOStk12 (MOI = 5 pfu/cell), or HSV-2(333)gJ- (MOI = 1.5 pfu/cell) for 5h at 37°C. Cells were lysed with 0.5% NP40 and β-galactosidase activity was measured by adding chlorophenol-red-β-D-galactopyranoside substrate and reading absorbance at 595 nm over 1h. For plaque assays, B78H1 cells in 24-well plates were stimulated with saliva proteins (2.5 mg/ml) or mock-treated with PBS and then exposed to HSV-1 KOS (10^4^ plaque forming units (pfu)) for 1h at 37°C. Inoculum was removed and cells were overlaid with medium containing 0.5% methylcellulose. After 48h, cells were fixed with methanol-acetone (2:1) and stained with anti-HSV antibodies using a black plaque assay [57]. Stimulation of AG09319 fibroblasts with *Porphyromonas gingivalis* lipopolysaccharides (LPS) (Sigma-Aldrich) was performed at concentrations up to 10 μg/ml [58] for 48h, prior to HSV-1 KOStk12 infection (MOI = 2) as described above.

### Virus titration

AG09319 fibroblasts were seeded in 96 well plates and stimulated with saliva proteins (donor 2; 4 mg/ml) for 48 hours. Medium was removed and cells were exposed to HSV-1 KOS (MOI = 5) in 50 μl at 37°C for 1h. Inoculum was removed, cells were washed with medium and 100 μl of fresh medium was added. At 8, 24 and 48 h, supernatant was collected and stored at -80°C. Titration was performed on Vero cells in 24-well plates. Serial dilutions of supernatant were prepared and 0.5 ml was added to duplicate wells. Inoculum was removed after 1 h at 37°C and cells were overlaid with medium containing 0.5% methylcellulose. After 72 h, the overlay was removed and cells were stained with 0.1% crystal violet in 25% methanol for 10 minutes before being washed and dried. Plaques were counted for at least 2 dilutions in duplicate to determine titers.

## Results

### Effect of salivary proteins on HSV-1 infection of diploid gingival fibroblasts

We used diploid human gingival fibroblasts (AG09319) as models for cells that may become exposed to HSV-1 and saliva after epithelium wounding or in periodontitis lesions. To address the effect of saliva on the susceptibility of gingival fibroblasts to HSV-1 infection, we exposed cells to semi-purified salivary components. Unstimulated saliva (i.e. collected by expectorating without mechanical/pharmacological stimulation) was clarified and filter-sterilized. The high-molecular weight fraction (>30 kDa) of each sample was used since the filtrate containing low-molecular weight molecules showed no detectable effect on HSV-1 infection. The range of protein concentrations used in our assays (0.05–5 mg/ml) encompasses the natural concentration of proteins in whole saliva (1–1.5 mg/ml) [45].

**Fig 1:**
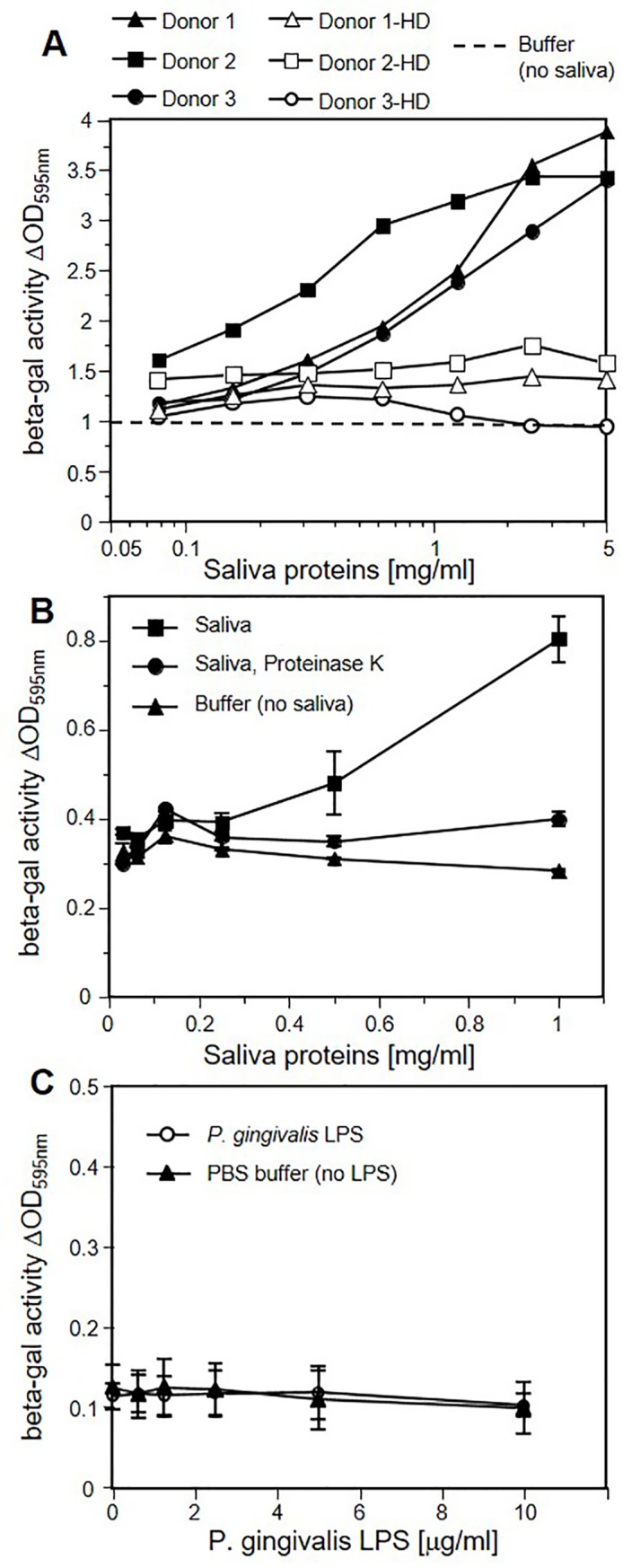
Human salivary proteins enhance HSV-1 infection of human gingival fibroblasts. (A) Infection assay of HSV-1 KOStk12, measured as activity of virus-encoded β-galactosidase. Saliva samples (30K retentate) from the indicated donors were added into the growth medium of AG09319 gingival fibroblasts for 48 h at the indicated concentrations. Growth medium containing salivary proteins was removed and cells were exposed to the reporter virus HSV-1 KOStk12 (MOI = 5) for 5 h. Cells were lysed, and the activity of β-galactosidase produced by the virus was measured to monitor infection. Black symbols represent native salivary samples. Open symbols represent heat-denatured (HD) saliva samples. The dashed line represents the baseline level of β-galactosidase activity in infected fibroblasts in the absence of saliva stimulation. Although saliva stimulation is consistently observed, the extent of the enhancement varied between experiments. Furthermore, the background levels of infection of AG09319 fibroblasts varied as well. The cause of this variation remains unclear. Thus, normalization of effect over background has proven to be uninformative in comparing experiments. Therefore, we show a representative of 2 or more experiments. (B) Saliva samples (30K retentate) from donor 4 were incubated with Proteinase K agarose for 4 h at 37°C or mock treated, and then serially diluted in culture medium for cell stimulation. The x axis is labeled according to protein concentration prior to PK treatment. AG09319 fibroblasts were treated and infected as in panel A. As a control without saliva stimulation, fibroblasts were mock treated with buffer (triangles). Error bars represent standard deviations between at least 3 independent experiments. (C) AG09319 gingival fibroblasts were pretreated with *P*. *gingivalis* LPS at the indicated concentrations prior to infection with HSV-1 KOStk12 (MOI = 2). An average of three experiments is shown. Error bars represent standard deviations. For comparison, infection levels in the absence of any stimulation, which are shown as a dashed line (panel A) or black triangles (panels B-C), correspond to equivalent “mock treatment” conditions.

We incubated AG09319 gingival fibroblasts with salivary samples (30 kDa retentate) for 48h, then exposed them to the reporter virus HSV-1 KOStk12 and measured β-galactosidase activity to monitor virus entry. We observed a dose-dependent enhancement of infection of AG09319 fibroblasts as the amount of saliva proteins from three donors was increased (Fig 1A, black symbols). The enhancing effect was lost upon boiling saliva (Fig 1A, open symbols). Because heat denaturation affects proteins more than glycosaminoglycans and other carbohydrates, this result suggests that the active factor may consist in, or at least contain, one or several proteins. We therefore treated saliva samples with proteinase K prior to stimulation of fibroblasts (Fig 1B). Proteinase K treatment greatly reduced saliva infection-enhancing ability, thus supporting the idea that the active factor comprised proteins.

Interestingly, susceptibility of oral fibroblasts to Kaposi sarcoma herpesvirus (KSHV) infection increased after cells were stimulated with lipoteichoic acid (LTA) or lipopolysaccharides (LPS) from *Porphyromonas gingivalis*, one of the bacteria causing periodontal disease [58]. To determine whether the same mechanism was involved in enhancing susceptibility to HSV-1 infection, we incubated AG09319 fibroblasts with various concentrations of *P*. *gingivalis* LPS for 48h. We then compared levels of infection between LPS-stimulated and mock-stimulated cells (Fig 1C). No increase of HSV-1 infection was observed in LPS-stimulated fibroblasts at concentrations that effectively enhanced cell susceptibility to KSHV [58]. This suggests that *P*. *gingivalis* LPS is unable to stimulate the cellular response required to enhance susceptibility to HSV-1. We cannot completely rule out the possibility that this inactivity may be due to a defect of the purified LPS reagent used here.

**Fig 2:**
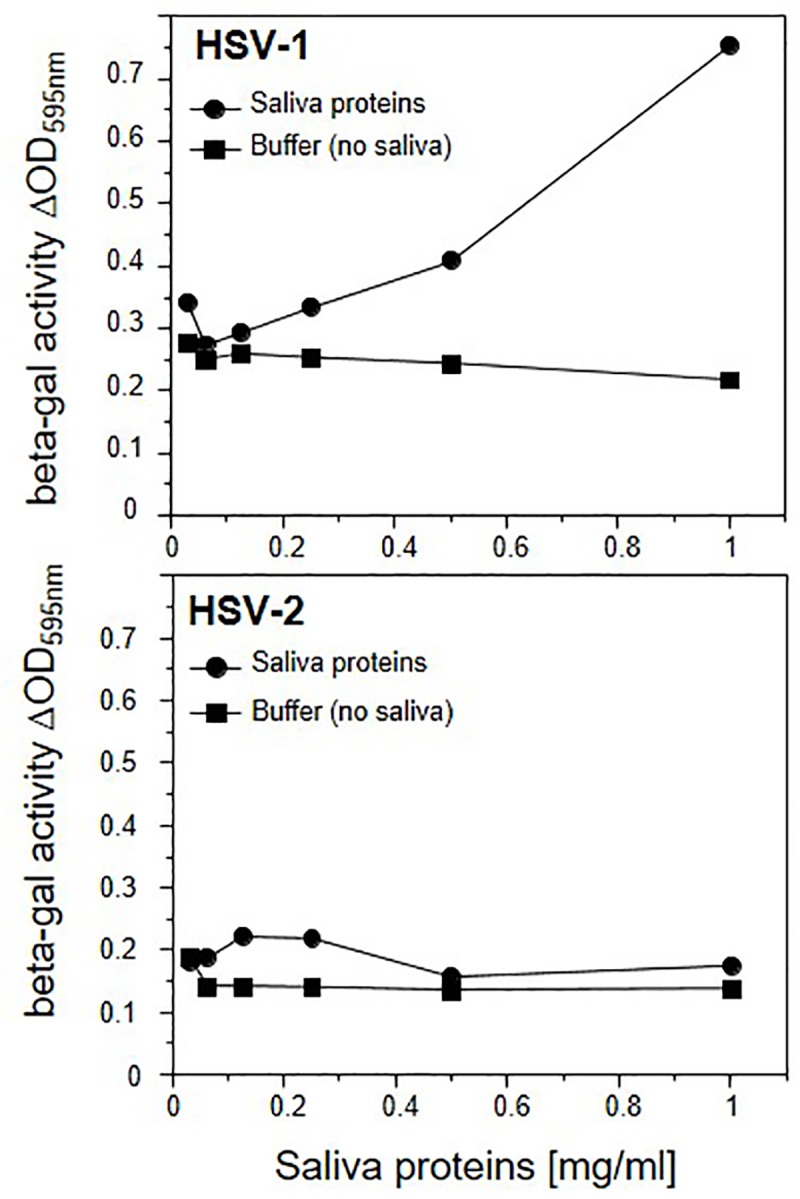
Human saliva enhances infection of human gingival fibroblasts by HSV-1 but not HSV-2. Diploid human oral fibroblasts AG09319 were exposed to salivary proteins (donor 1) at the indicated concentrations or mock treated with PBS (buffer), and exposed to HSV-1 KOStk12 or HSV-2(333)gJ- reporter viruses at MOIs of 5 and 1.5 pfu/cell respectively. The activity of virus-encoded β-galactosidase is used to monitor infection. A representative of at least three experiments is shown.

We then compared the effect of salivary proteins on the susceptibility of gingival fibroblasts to HSV-1 and HSV-2, which is not a frequent oral pathogen. We used two *lacZ* reporter viruses: HSV-1 KOStk12 and HSV-2(333)gJ- [50–52]. Both recombinant viruses infected AG09319 fibroblasts efficiently, as tested by β-galactosidase activity (S1 Fig). The difference in efficacy of infection likely reflects biological differences between the two viruses. However, since β-galactose expression is driven by two different promoters in these constructs [50–52], it is also possible that the level of expression contributes to the observed difference. Based on these data, we performed stimulation experiments using an MOI of 1.5 for HSV-2(333)gJ-, which generates a signal comparable to that of HSV-1 KOStk12 at an MOI of 5 pfu/cell (S1 Fig). Cells pre-incubated with salivary extracts showed a dose dependent increase in susceptibility to HSV-1 KOStk12 but not HSV-2(333)gJ- infection (Fig 2). Thus, the saliva-induced response of oral fibroblasts appears to distinctively affect the oral pathogen HSV-1.

**Fig 3:**
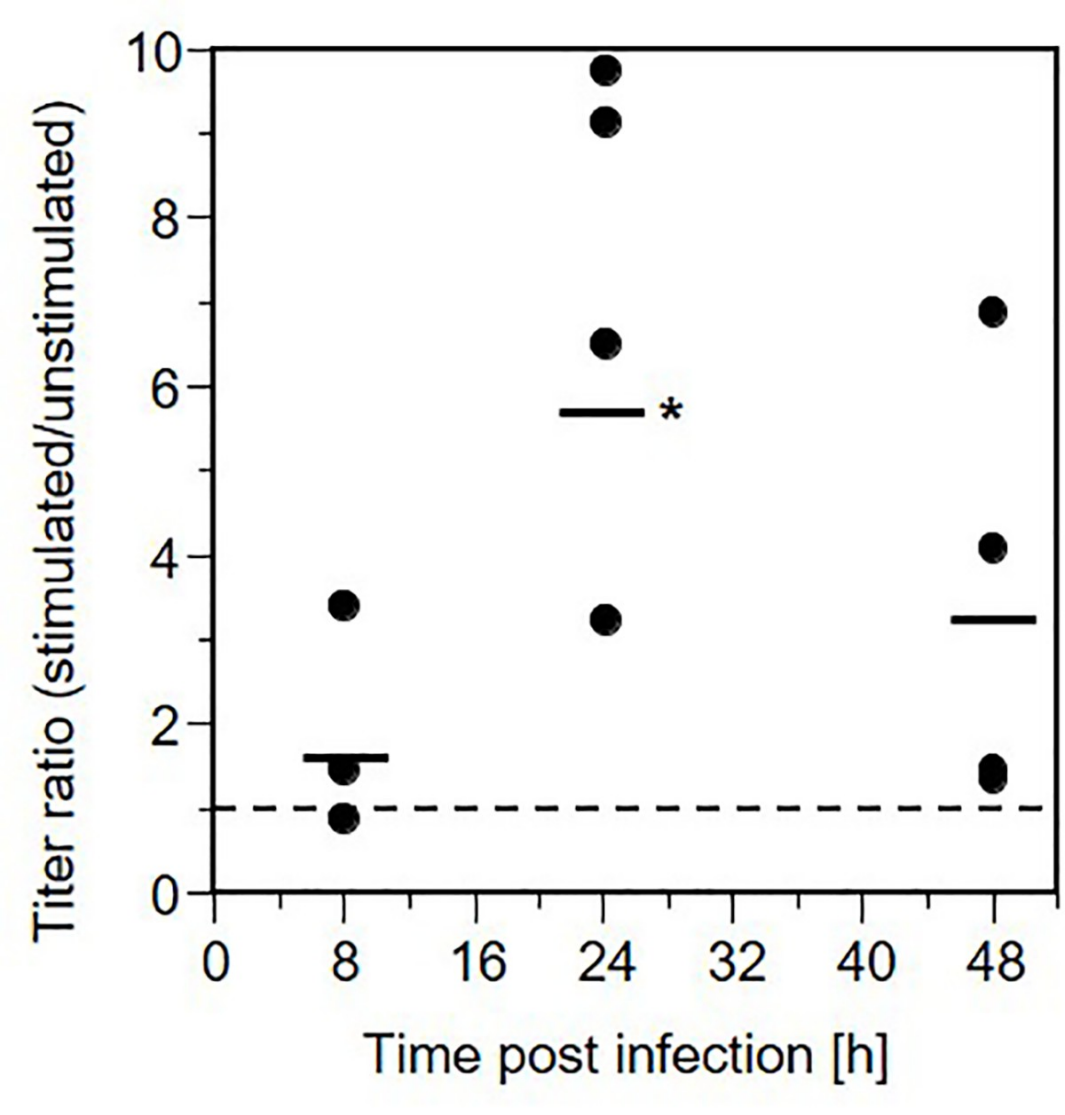
HSV-1 production by gingival fibroblasts stimulated by human saliva. AG09319 fibroblasts were stimulated with saliva proteins (donor 2) at 4 mg/ml for 48 h prior to infection. Cell culture supernatants were collected at 8, 24 and 48 h post-infection and titered on Vero cells. Each dot represents the ratio of titers of stimulated over unstimulated cells. A ratio of 1 (dashed lines) corresponds to similar titers in stimulated and control cells. Data from four stimulation experiments are shown. The horizontal bars indicate average fold increase. Statistical t-test analysis showed that the average increase in titer in stimulated cells is statistically significant at 24 h post-infection (p = 0.0169), indicated by an asterisk.

The effect of saliva stimulation on HSV-1 production by AG09319 gingival fibroblasts was assessed by determining viral titers in culture medium at various times after infection (Fig 3). At 8 h post-infection (p.i.), viral titers were not significantly different between stimulated and unstimulated cells, although cytopathic effects appeared more pronounced in saliva-stimulated cells. At 24 h p.i., virus titers were 3 to 10-fold higher in stimulated cells compared to unstimulated cells. Although a large variability was observed, this increase was significant (p = 0.0169). After 48 h, titers from stimulated cells remained higher but were not significantly different. This suggests that saliva stimulation led to an increased production of infectious virus between 8 and 24 h after infection.

### Effect of saliva on cells resistant to HSV infection

**Fig 4:**
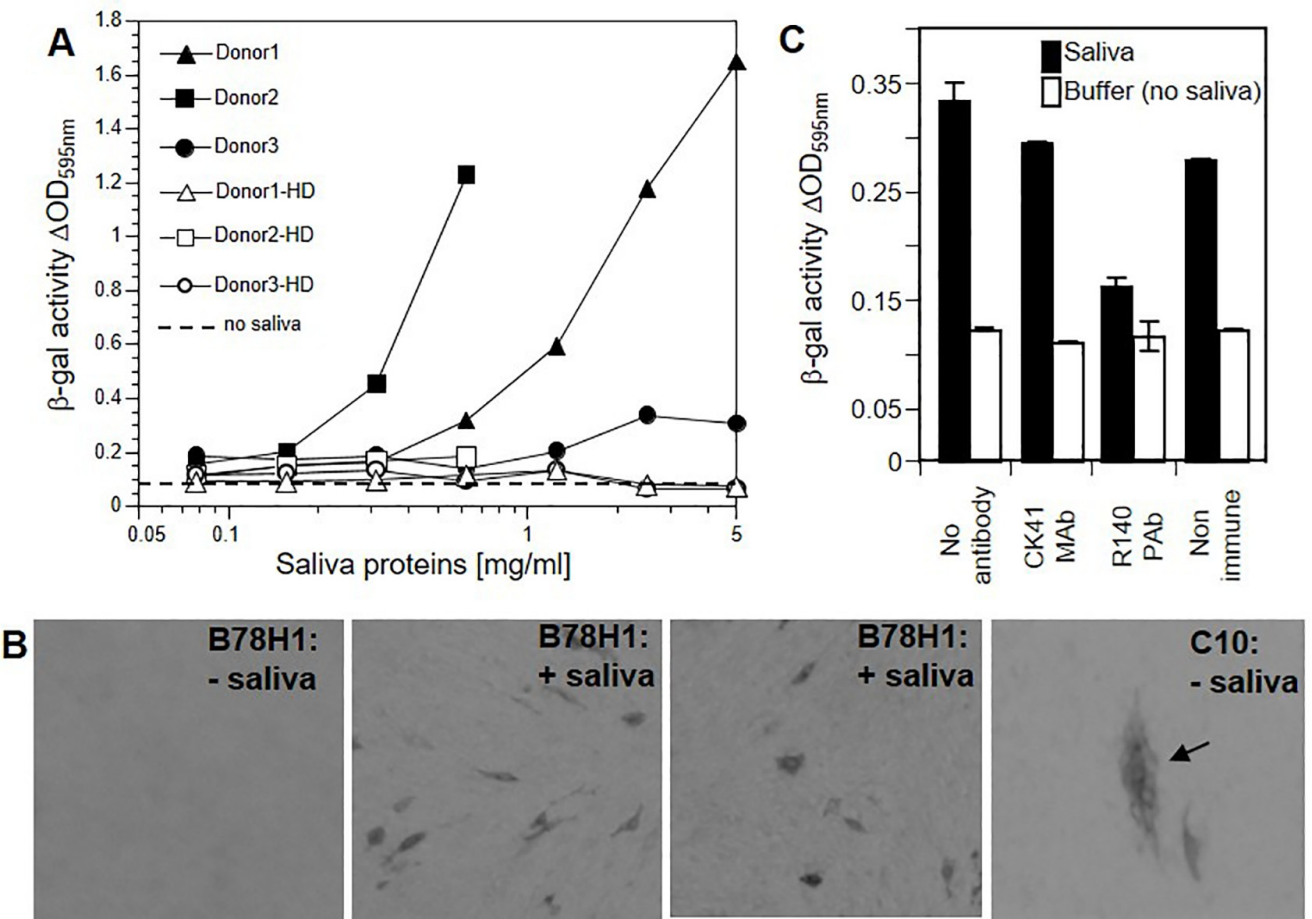
Human saliva promoted entry into receptor deficient B78H1 cells. (A) Entry assay. Salivary proteins (30K retentate) from the indicated donors were added into the growth medium of B78H1 cells for 48 h. Growth medium containing salivary proteins was removed and cells were exposed to HSV-1 KOStk12 for 5 h. Cells were lysed, and β-galactosidase activity was measured to monitor infection. Black symbols represent native saliva proteins. Open symbols represent saliva proteins which were heat-denatured (HD) by boiling for 5 min. The dashed line represents the baseline level of β-galactosidase activity in infected B78H1 cells in the absence of saliva stimulation. (B) Plaque assay. B78H1 cells were pre-treated with saliva proteins (donor 1) for 48 h. Medium was removed and cells were exposed to 10^4^ pfu of HSV-1 KOS. Cells were overlaid with methylcellulose and plaque were allowed to form for 48 hours. Infected cells were immunostained with anti-HSV glycoprotein antibodies. As a control for infection, susceptible C10 cells (i.e. B78H1 cells expressing human nectin-1) were exposed to only 10^2^ pfu of HSV-1 KOS and showed plaques comprising multiple infected cells (arrow, right panel). The left panel shows unstimulated B78H1 cells and no signs of infection. (C) Entry assay in the presence of anti-receptor antibodies that specifically block usage of nectin-1 (monoclonal antibody CK41) or HVEM (polyclonal serum R140). B78H1 cells were stimulated (black) or not (white) with saliva proteins from donor 2 (30K retentate, 1.8 mg/ml). After removal of saliva, cells were preincubated with purified immunoglobulins from R140 serum or CK41 monoclonal antibody at 100 mg/ml for 1 h before the addition of HSV-1 KOStk12. Non-immune immunoglobulins were used as control. The activity of virus-encoded β-galactosidase is used to monitor infection. An average of three experiments is shown with error bars representing standard deviations.

Since saliva extracts from different donors enhanced infection of gingival fibroblasts that are susceptible to HSV-1, we asked whether they could also allow infection of resistant cells. We previously characterized mouse melanoma B78H1 cells, which are resistant to infection by HSV-1 and HSV-2 due to the absence of a functional entry receptor but become fully permissive upon expression of human HVEM or nectin-1[49, 59]. We stimulated B78H1 cells with saliva for 48 h and exposed them to HSV-1 KOStk12. Surprisingly, robust infection was observed following exposure to saliva proteins (Fig 4A). As seen for gingival fibroblasts, heat-treatment abolished the stimulatory effect of saliva (Fig 4A). Interestingly, although saliva from donors 1 and 3 were equally effective in enhancing infection of oral fibroblasts (Fig 1A), saliva from donor 3 was less efficient in stimulating infection of B78H1 cells (Fig 4A). The different sensitivity of these cell types to stimulation alone does not explain this discrepancy. It is possible that a variation in the nature or amount of the stimulating factor in donor 3 saliva may account for this peculiarity. A systematic comparison of donors from a larger cohort may help define accurately such differences between individuals. Because unstimulated B78H1 cells are completely resistant to HSV-1, we could also use a classical plaque formation assay to assess infection after saliva stimulation. Untreated B78H1 cells showed no sign of infection after exposure to HSV-1 (Fig 4B, left). In contrast, infection of control C10 cells (B78H1 cells expressing human nectin-1) yielded multicellular plaques (Fig 4B, right). Interestingly, many B78H1 cells stimulated with saliva proteins became infected (Fig 4B, center). Only individual infected cells were visible and plaques that involved multiple cells were not seen. Although it is possible that saliva stimulation allowed infection by free virions but not cell-to-cell spread, a likely explanation is that only a subset of B78H1 cells responded sufficiently in order to become susceptible to infection. The limited number of responsive cells would prevent plaque formation. Further studies will be necessary to determine and quantify the activated cellular responses in order to dissect possible differences between susceptibility to entry and spread after saliva stimulation.

Next, we investigated whether HSV-1 entry into saliva-stimulated B78H1 cells followed the natural receptor-mediated pathway. We used well-characterized blocking antibodies R140 and CK41 to prevent usage of HVEM and nectin-1 respectively [54, 55]. HSV-1 entry into stimulated B78H1 cells was efficiently blocked by the anti-HVEM pAb R140, but not with the anti-nectin-1 mAb CK41 (Fig 4C). This suggests that saliva somehow stimulated surface expression of HVEM in B78H1 cells to allow entry. Immunofluorescence assays did not detect an increase of HVEM surface expression in saliva stimulated cells. However, B78H1 cells, which expressed receptors below levels of detection were previously shown to be susceptible to infection by HSV-1 KOStk12 [37]. Overall our data obtained in B78H1 cells indicate that human salivary proteins can expand HSV-1 tropism to resistant cells by enhancing virus entry.

### Effect of saliva from different donors

**Fig 5:**
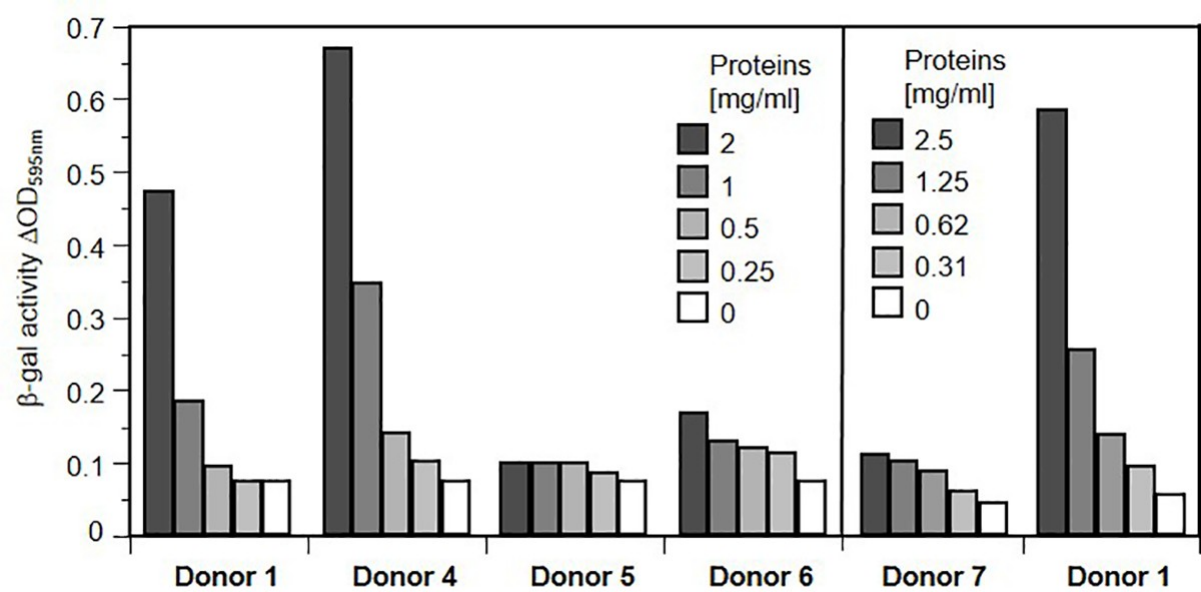
Variation and dose response of salivary activity between donors. Salivary proteins (30 kDa retentate) was added to B78H1 cell culture medium at the indicated concentrations for 48 h. Stimulating medium was removed and cells were exposed to HSV-1 KOStk12 for an entry assay. Infection was monitored by measuring β-galactosidase activity after a 5 h infection. Results from a representative of at least two independent experiments is shown.

Since we observed marked differences in the ability of saliva from different donors to stimulate HSV-1 infection of gingival fibroblasts and B78H1 cell line (Figs 1A and 3A), we directly compared samples from additional donors (Fig 5). While donors 1 and 4 displayed high stimulation ability, donors 6 and 7 had a much lower activity and donor 5 had no detectable activity above experimental background. This study does not include longitudinal follow-ups of donors, but when several batches from the same donor (taken over several months) could be tested, stimulation effectiveness appeared consistent between donations. Donors were required not to provide samples if they exhibited herpes or periodontal lesions (based on personal observation). The limited cohort of donors enrolled in this study to test the effect of saliva on infection of cultured cells does not allow for establishing a correlation between the unexpected variability in saliva activity and serological HSV-1 and -2 status. Nevertheless, at least one donor (donor 2) was seronegative for HSV-1 and -2 during the study and yet had a highly active saliva. This limited observation suggests that the infection-enhancing effect in saliva is not induced by prior infection by HSV.

### Effect of different saliva fractions

**Fig 6:**
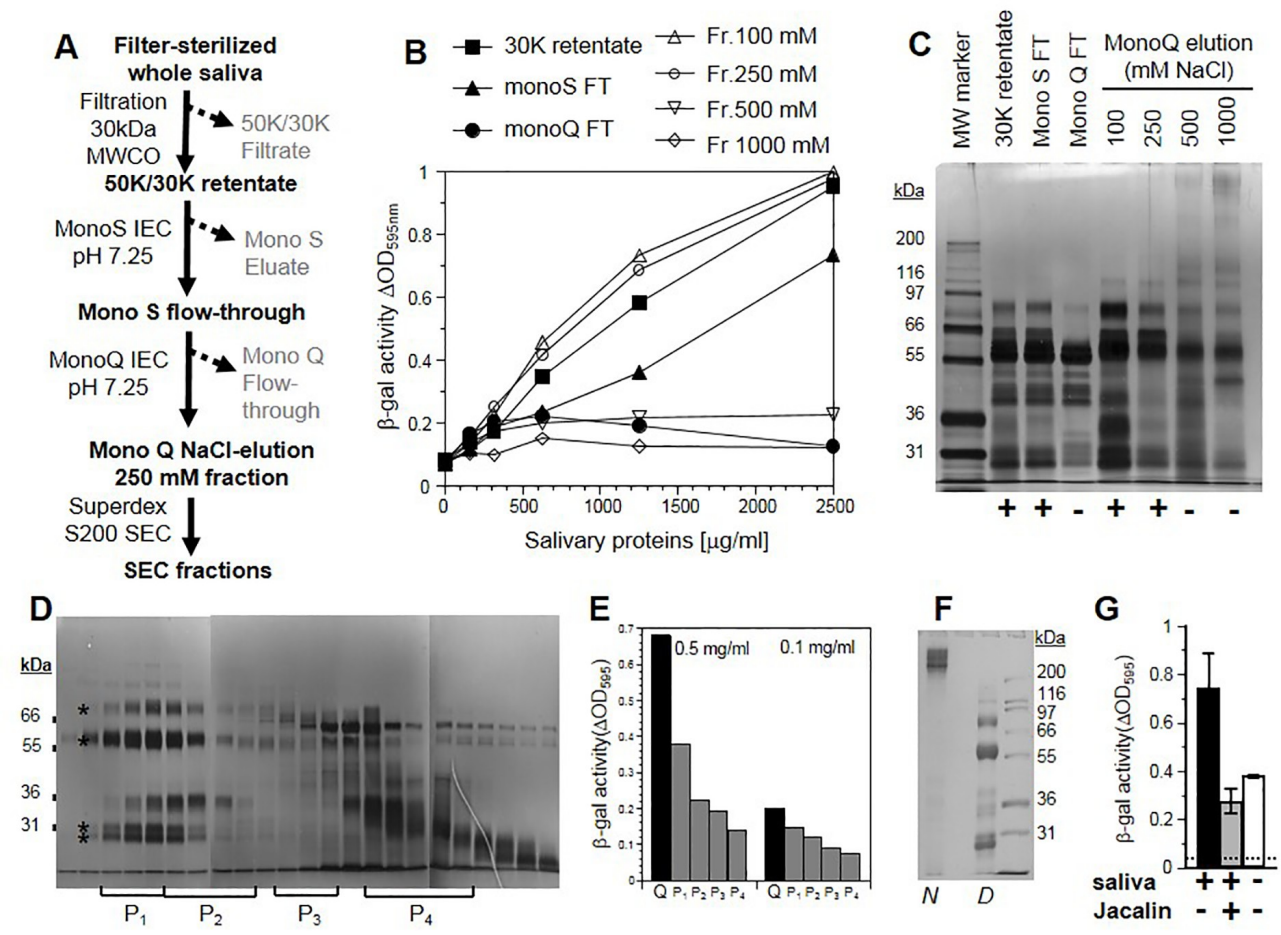
Partial fractionation of salivary components. (A) Diagram of fractionation steps through cation (Mono S) and anion (mono Q) exchange chromatography. FT: Flow-through. (B) Saliva activity determined by a virus entry assay. B78H1 cells were incubated in the presence of various concentrations of salivary proteins (donor 1) from the indicated fractions during 48 h. Saliva-containing growth medium was removed and cells were exposed to HSV-1 KOStk12 for 5 h. Infection was monitored by β-galactosidase activity by measuring absorption of substrate (CPRG) at 595 nm. A representative of at least two experiments is shown. (C) Silver stained gel showing proteins in the indicated fractions. + and–signs indicate the presence of infection-enhancing activity as determined in C. Molecular weights (MW) of marker proteins are indicated. (D) Size exclusion chromatography. Fraction eluted from the monoQ column with 250mM NaCl was loaded on a Superdex200 column. Proteins from size exclusion fractions were subjected to SDS-PAGE. A composite image of silver stained gels from the same experiment is shown. P_1_ to P_4_ indicate pooled fractions used in stimulation assay. (E) Elution fractions were pooled (P_1_ to P_4_), proteins were quantified and used at two concentrations to stimulate B78H1 cells. Infection by HSV-1 KOStk12 is reported as β-galactosidase activity as in B. A representative of at least 2 experiments is shown. (F) Fractions from activity peak 1 (panel D and E) were pooled and loaded on a polyacrylamide gel under native (*N*) or denaturing conditions (*D*). (G) Unfractionated saliva sample (30 kDa retentate) was incubated with Jacalin-agarose for 4 h at 4°C. Treated and untreated salivary proteins were tested on AG09319 fibroblasts. After 48 h pre-treatment with salivary proteins (1 mg/ml) cells were infected with HSV-1 KOStk12. Infection is reported as β-galactosidase activity 5 h post-infection. The dotted line indicates background signal in the absence of infection. Error bars represent standard deviations.

To begin to address the nature of the active protein(s), we fractionated saliva components using ion-exchange (IEC) and size-exclusion chromatography (SEC) (Fig 6A). Activity was recovered in the flow-through after passing through a cation-exchange MonoS column (Fig 6B). We then loaded this flow-through material onto an anion-exchange MonoQ column. In that case, the active compounds were bound and the flow-through was devoid of activity (Fig 6B). A NaCl concentration gradient was used to elute bound materials. Most activity was recovered in fractions between 100 and 250 mM NaCl (Fig 6B). The protein profiles of all samples are shown in Fig 6C. We further separated components of the 250 mM NaCl fraction on the basis of molecular weight using a superdex200 column (Fig 6D). To obtain sufficient material for cell stimulation, fractions were pooled. Although some activity was lost by this purification protocol, we nevertheless, recovered most of the remaining activity in early fractions (pool 1) comprising high molecular weight molecules (Fig 6E).

Using non-denaturing PAGE [60], we observed that the active fractions indeed contained protein complexes with high molecular weights, while under denaturing conditions, low molecular weight bands were seen (Fig 6F). The size of several proteins appeared consistent with polypeptides of immunoglobulins. To address the possible involvement of salivary Immunoglobulin A (IgA), an unfractionated saliva sample was incubated with jacalin (a lectin with high affinity for IgA). After IgA-depletion, saliva samples failed to stimulate HSV-1 infection of gingival fibroblasts (Fig 6G). This suggests that IgA or other glycoproteins bound by jacalin may be involved. Altogether these data show that the active factor comprises high-molecular weight glycoprotein complexes, which may include non-HSV induced Immunoglobulin A.

## Discussion

Here we report that a human salivary factor has the ability to enhance susceptibility of human gingival fibroblasts to HSV-1. Saliva is well known to have antiviral effects and inactivate HSV-1 virions [45–47], it was surprising to find that saliva effect on cells can enhance susceptibility to HSV-1 infection. This observation brings up several questions. First, what are the cellular responses that affect susceptibility to infection? Second, what is the origin of the active components in certain saliva? Third, does this activity influence the probability of acquiring HSV-1, the severity of recurring oral herpes or the possible role of HSV-1 in periodontal disease? This study does not definitively answer these questions, but provides a new vantage point to address the role of saliva in HSV-1 infection.

Susceptibility of cells to viral infections is determined by the combination of a number of parameters that are difficult to assess independently. Here, incubation with saliva extracts modulates the susceptibility of gingival fibroblasts to HSV-1 infection. This stimulation also led to a transient but significant increase in viral production. When a high MOI was used to infect AG09319 cells *in vitro*, we observed a 3 to 10-fold increase of titers at 24 h post infection. Whether this enhanced viral production directly affects the pathogenesis of HSV-1 or periodontal disease *in vivo* remains to be seen. The infection-enhancing effect is observed after several hours of stimulation suggesting that the response may involve changes in gene expression. Indeed a similar observation was made regarding changes in susceptibility of oral fibroblasts to Kaposi sarcoma herpesvirus (KSHV) [58]. KSHV infection of oral fibroblasts increased only after a 24 h stimulation by lipoteichoic acid (LTA) or lipopolysaccharides (LPS) from *Porphyromonas gingivalis*, one of the main causes of periodontitis. Our data showed that similar concentrations of *P*. *gingivalis* LPS were ineffective in increasing HSV-1 infection of oral fibroblasts and did not mimic the enhancing effect of salivary proteins (Fig 1C). It is unlikely that the same mechanism leads to enhanced susceptibility to KSHV and HSV-1 infections in response to two different stimuli. Nevertheless, we do not exclude the possibility that overlapping signaling pathways may be involved. In the case of KSHV, an NFκB cascade was activated by LPS, however it is unclear how this response enhanced cell susceptibility to infection [58]. NFκB activation in response to LPS is well documented [61, 62] but data on signal transduction pathways and changes in gene expression in response to saliva’s multiple components are limited [63, 64]. Cell susceptibility to HSV-1 infection is multifactorial but is strongly influenced by the number of entry receptors. Availability of nectin-1 or HVEM correlates directly with HSV-1 infection efficiency in cell lines, cancer cells and tissues [37, 38, 65]. Since saliva stimulation allowed infection of receptor-deficient B78H1 cells, we used neutralizing antibodies against HVEM and nectin-1 to determine if these receptors are used *de novo*. Whereas anti-nectin-1 mAb CK41 had no detectable effect, an anti-HVEM serum prevented infection, indicating that newly available HVEM may allow virus entry into saliva-stimulated B78H1 cells. Regulation of HVEM expression has not been analyzed but it is known to respond to cellular changes, for instance during lymphocyte activation [66, 67]. The situation is more complex in AG09319 and other oral fibroblasts since both HVEM and nectin-1 are present and used by HSV-1 and -2 to enter these cells [23, 24, 68]. In fact, blocking a single receptor with antibodies does not provide reliable information on receptor activity in fibroblasts because of possible compensatory use of the alternate receptor [37]. Although stimulation of B78H1 cells show that saliva can expand infection of HSV-1 to normally resistant cells, it does not imply that HVEM availability is the only factor that modulates susceptibility of gingival fibroblasts. Additional elements likely play a role since saliva exposure increased infection of gingival fibroblasts by HSV-1 but not HSV-2. Since HVEM is a functional receptor for both HSV-1 and -2 [51, 55], a different part of the response may favor HSV-1 over HSV-2. This study takes advantage of reporter viruses which have frequently been used as representatives of each type. We note that although HSV-2(333)gJ- uses HVEM and nectin-1, this reporter virus had a decreased ability to use an unidentified gD receptor expressed endogenously in CHO cells [50]. This characteristic is unlikely to be the cause of the inability of this virus to better enter saliva-stimulated fibroblasts. However, it is clear that reporter strains only offer a very limited view of the variation among HSV-1 and HSV-2 clinical isolates [37]. Oral herpes is overwhelmingly caused by HSV-1 compared to HSV-2, unlike genital herpes which is caused by either viruses [3, 69]. The differences in distribution of HSV-1 and HSV-2 may be due to a combination of physiological and cellular factors, viral determinants, and behavioral practices. Change in oral-genital sexual practices has been proposed as a reason for the increased proportion of genital herpes cases caused by HSV-1 [70]. Nevertheless, the cases of oral herpes caused by HSV-2 remain rare [3]. Cellular or physiological causes, which may affect the distribution of HSV-1 and HSV-2 remain unknown. While remaining cautious, it is attractive to think that the characterization of this new HSV-1-specific response of oral cells to saliva may provide some clue to understanding the predominance of HSV-1 in oral infections.

Saliva is a very complex fluid with components derived from genetic, immunological and microbial origins [40, 48, 71]. All three origins contribute to individual variability and could be the source of the infection-enhancing activity. Genetic factors are well known to affect susceptibility to viruses and other pathogens. However, there are no known genetic markers linked to HSV-1 oral infection. In children, inborn errors in interferon production in neurons are linked to increased risk of HSV encephalitis [9]. Pinpointing a possible genetic factor responsible for the enhancing activity will require a large cohort of individuals and the analysis of parotid, submandibular and sublingual saliva devoid of microbial products. Immune factors such as salivary IgA vary in specificity and quantity between individuals [72]. Our data suggest an involvement of salivary IgA and the variability between donors may reflect their global immunological history. Antibody-dependent enhancement (ADE) of infection has been described for several viruses, including HSV-1 [73, 74]. ADE requires that virions become coated with antiviral immunoglobulin. Here however, saliva acts on cells, not on virions. Furthermore, ADE involves antibodies against the specific pathogen. Here, donor 2 is seronegative for HSV-1 and 2 but has potent infection-enhancing saliva. This suggests that anti-HSV IgA were not involved in enhancing HSV-1 infection. The involvement of cross-reacting IgA from unknown origin cannot be excluded, since cross-reacting antibodies are known to mediate ADE, for instance for dengue and Zika viruses [75]. The oral microbiome (>600 species) plays a critical role in host health and diseases [76]. Products from the oral microflora may act to increase HSV-1 susceptibility of oral fibroblasts. We found that *P*. *gingivalis* LPS which increased susceptibility of such cells to KSHV infection [58] did not stimulate susceptibility to HSV-1 infection under similar conditions. We cannot exclude that proteinaceous byproducts from the microflora may be directly responsible for the HSV-1 infection-enhancing effect found in the saliva of certain donors. Furthermore, the composition of each individual’s microflora may elicit IgA responsible for the variation in infection-enhancing activity in saliva. The variability of the oral microbiome between individuals would be consistent with the variation of the effects seen between donors. However, a more comprehensive study will be necessary to correlate the presence of specific microbial products or cognate IgA with the saliva activity that increases cell susceptibility to HSV-1.

In our small cohort, we found donors with highly potent saliva and others with very low or undetectable activity. However, our observations suggest that it is not necessary to be HSV seropositive to display this activity (e.g. donor 2). It is nevertheless possible that the presence of the active salivary factor(s) may cause certain individuals to be more at risk of acquiring oral HSV-1 infection, have more severe symptomatic reactivation events and/or shed more virus. Clearly this infection-enhancing activity is not a marker for HSV infection. However, it may become associated with an increased risk for infection if future data were to show that carriers of this activity are more frequently infected by HSV-1 orally. Also, this salivary factor may influence the impact of HSV-1 on periodontal disease since it acts on oral fibroblasts. Again, one can speculate that the presence of HSV-1 in PD lesions may be more frequent in individuals who display the infection enhancing activity in saliva. The frequency of this salivary activity in the population is unknown and cannot be extrapolated from the small sample size from our study, which was not designed to collect epidemiological data. A more complete epidemiological study should determine whether this salivary effect can be used as a biomarker or risk factor for oral herpes susceptibility or severity. To fully understand the role of saliva on HSV-1 oral pathogenesis, the contribution of the salivary factors which enhance cell susceptibility to HSV-1 infection must be considered in conjunction with the action of anti-HSV-1 factors found in saliva.

## Supporting information

S1 Fig Infection of gingival fibroblasts with reporter viruses. AG09319 fibroblasts were infected with *LacZ* recombinant viruses HSV-1 KOStk12 and HSV-2(333)gJ- at the indicated MOI. After 6 hours, cells lysed with 0.5% NP40 and b-galactosidase activity was measured by adding chlorophenol-red-β-D-galactopyranoside substrate and reading absorbance at 595 nm over 1 h. A representative of 2 experiments is shown. Error bars represent standard deviations of duplicates.
(TIF)
