# Supplementary figures and images for: Saliva enhances infection of gingival fibroblasts by herpes simplex virus 1
Source: PLoS One. 2019 Oct 3;14(10):e0223299. doi: 10.1371/journal.pone.0223299 (PMC6776388; doi:10.1371/journal.pone.0223299)

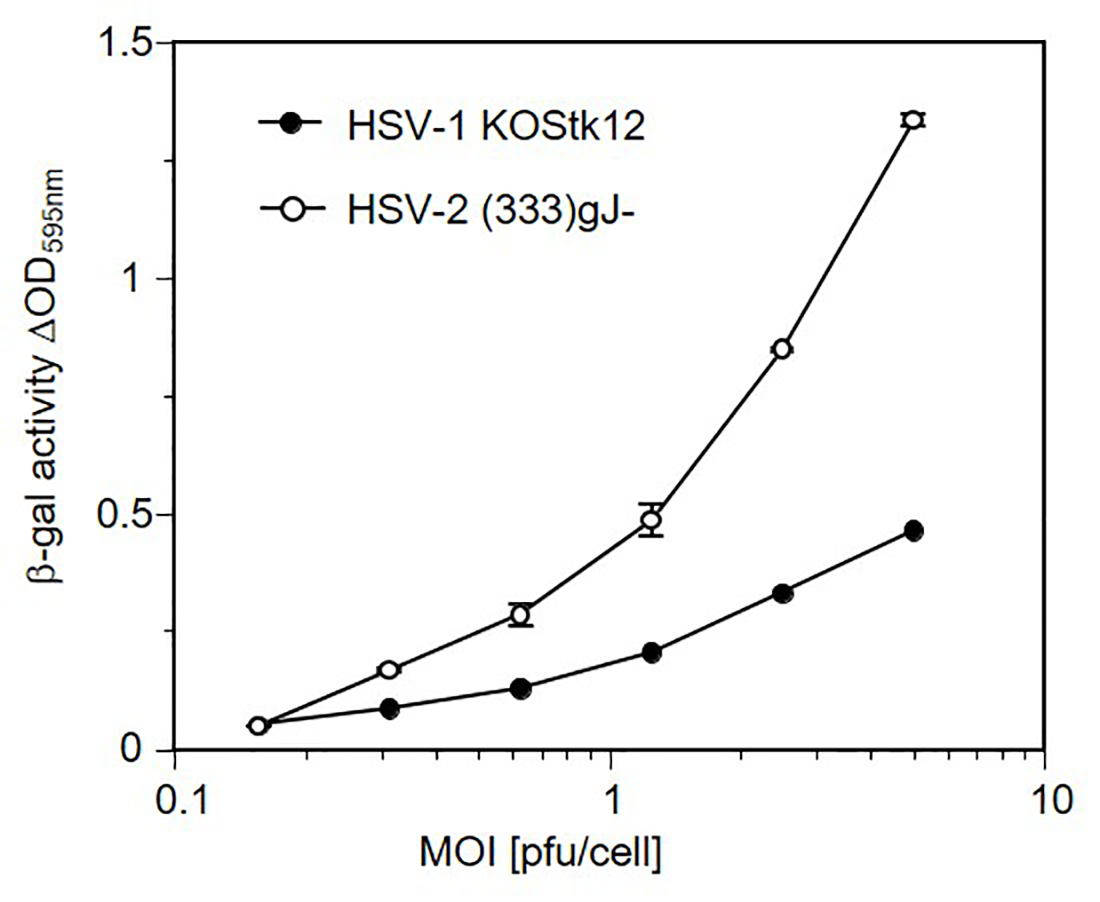

Supplement: S1 Fig — AG09319 fibroblasts were infected with LacZ recombinant viruses HSV-1 KOStk12 and HSV-2(333)gJ- at the indicated MOI. After 6 hours, cells lysed with 0.5% NP40 and b-galactosidase activity was measured by adding chlorophenol-red-β-D-galactopyranoside substrate and reading absorbance at 595 nm over 1 h. A representative of 2 experiments is shown. Error bars represent standard deviations of duplicates. (TIF) [file pone.0223299.s001.tif]
